# Identification of HSP47 Binding Site on Native Collagen and Its Implications for the Development of HSP47 Inhibitors

**DOI:** 10.3390/biom11070983

**Published:** 2021-07-03

**Authors:** Haiyan Cai, Parvathy Sasikumar, Gemma Little, Dominique Bihan, Samir W. Hamaia, Aiwu Zhou, Jonathan M. Gibbins, Richard W. Farndale

**Affiliations:** 1 Key Laboratory of Cell Differentiation and Apoptosis of Chinese Ministry of Education, Department of Pathophysiology, Shanghai JiaoTong University School of Medicine, Shanghai 200025, China; hycai@shsmu.edu.cn (H.C.); awz20@shsmu.edu.cn (A.Z.); 2 Institute for Cardiovascular & Metabolic Research, School of Biological Sciences, University of Reading, Health and Life Sciences Building, Whiteknights, Reading RG6 6EX, UK; p.sasi-kumar@imperial.ac.uk (P.S.); gemma.little@reading.ac.uk (G.L.); j.m.gibbins@reading.ac.uk (J.M.G.); 3 Department of Biochemistry, University of Cambridge, Downing Site, Cambridge CB2 1QW, UK; dominique.bihan@ucalgary.ca (D.B.); swh23@cam.ac.uk (S.W.H.); 4 CambCol Laboratories Ltd., Ely CB6 1RS, UK

**Keywords:** HSP47 inhibitor, collagen, fibrosis, molecular docking, structural analysis

## Abstract

HSP47 (heat shock protein 47) is a collagen-specific molecular chaperone that is essential for procollagen folding and function. Previous studies have shown that HSP47 binding requires a critical Arg residue at the Y position of the (Gly-Xaa-Yaa) repeats of collagen; however, the exact binding sites of HSP47 on native collagens are not fully defined. To address this, we mapped the HSP47 binding sites on collagens through an ELISA binding assay using collagen toolkits, synthetic collagen peptides covering the entire amino acid sequences of collagen types II and III assembled in triple-helical conformation. Our results showed that HSP47 binds to only a few of the GXR motifs in collagen, with most of the HSP47 binding sites identified located near the N-terminal part of the triple-helical region. Molecular modelling and binding energy calculation indicated that residues flanking the key Arg in the collagen sequence also play an important role in defining the high-affinity HSP47 binding site of collagen. Based on this binding mode of HSP47 to collagen, virtual screening targeting both the Arg binding site and its neighboring area on the HSP47 surface, and a subsequent bioassay, we identified two novel compounds with blocking activity towards HSP47 binding of collagen. Overall, our study revealed the native HSP47 binding sites on collagen and provided novel information for the design of small-molecule inhibitors of HSP47.

## 1. Introduction

Collagen is the most abundant protein in mammals and is critical for forming specialized extracellular networks that bind cells together. The folding, processing, and assembly of collagen is tightly regulated in eukaryotic cells. Procollagen, the precursor molecule of collagen, undergoes extensive posttranslational processing to assemble into a triple-helical collagen, following which mature collagen is then secreted [1,2]. During these processes, several molecular chaperones and enzymes such as prolyl hydroxylase and heat shock protein 47 (HSP47) are involved. 

HSP47 is a member of the serpin family, but it lacks serine protease inhibitory activity [3]. It normally resides in the endoplasmic reticulum (ER) and functions as a collagen-specific molecular chaperone [4,5,6]. HSP47 associates transiently with procollagen in the ER, thereby preventing premature interactions between nascent procollagen molecules, and dissociates when procollagen transfers to cis-Golgi [7]. HSP47 knockout mice are embryonic-lethal 11.5 days post-coitus [8], and aberrant formation of triple-helical collagen I molecules as well as defects in collagen production and basement membrane formation have been detected in these embryos. This indicates that HSP47 is essential for the correct folding of procollagen.

Overexpression of HSP47 is associated with abnormal deposition of collagens in the extracellular matrix (ECM), with the onset of various fibroses, including liver cirrhosis, lung, bone marrow, and idiopathic pulmonary fibroses [9,10]. No specific treatment is currently available for these fibrotic diseases, which often lead to organ or tissue failure [11]. Nevertheless, the progression of fibrosis could be markedly reduced when HSP47 expression is suppressed using siRNA-mediated knockdown [12,13]. Altered HSP47 expression levels are also found to correlate with the development of several types of cancer, such as cervical, breast, pancreatic, and gastric cancers [13,14]. Silencing HSP47 significantly inhibits cell invasion in breast cancer cells and inhibited tumor growth in a xenograft mammary tumor model [4,15]. Our recent studies showed that HSP47 is present on the platelet surface and is involved in the recognition and response to collagen [16]. It is plausible that aberrant expression of HSP47 may contribute to the onset of various cardiovascular diseases. As HSP47 is the only collagen-specific molecular chaperone, targeting its binding interactions with collagen could be an effective therapeutic approach in managing these HSP47-overexpression-related diseases. Several types of small molecules have been developed as HSP47 inhibitors [17,18,19,20]. Recently, one of these compounds (COM IV) was reported to effectively prevent collagen synthesis, cell viability, and migration of lung fibroblasts [21].

There have been extensive biochemical and structural studies on the binding interactions between HSP47 and collagen using short synthetic collagen–mimetic peptides [7,22,23]. These showed that HSP47 binds to triple-helical peptides containing a key Arg residue at the Y position of collagenous repeats (Gly-Xaa-Yaa). These results were confirmed by X-ray crystal structures of collagen model peptides (designated CMPs) complexed with canine HSP47 [24]; however, the exact binding sites of HSP47 on native collagens are not fully defined. For example, there are more than 40 arginine residues at similar Y positions in collagen II or III, and it is unclear if HSP47 could bind them all. To clarify this, here we have mapped the HSP47 binding sites using collagen toolkits and identified HSP47 binding sites on collagens II and III. This provides novel information on the binding mode of HSP47 on collagen and sheds light on the development of novel HSP47 inhibitors. 

## 2. Materials and Methods 

### 2.1. Expression and Purification of HSP47 

Genes encoding human HSP47 (amino acids 19-418) were cloned in pET-28a. Proteins were expressed with C-terminal His-tag in *E. coli* BL21 (DE3). Cells were cultured at 37 °C in LB and induced with 0.5 mM IPTG (isopropyl-β-D-thiogalactoside) until A_600_ reached 1. After a further 16 h incubation at 25 °C, cells were collected by centrifugation, then resuspended in lysis buffer (20 mM Tris-HCl pH 7.4, 0.5 M NaCl) and lysed by high pressure. His-tagged HSP47 in the supernatant was isolated by nickel-affinity chromatography (GE Healthcare). The column was washed with wash buffer (20 mM Tris-HCl pH 7.4, 0.5 M NaCl, 20 mM imidazole) and step-eluted with elution buffer (20 mM Tris-HCl pH 7.4, 0.5 M NaCl, 300 mM imidazole). The peak fractions were pooled and dialyzed against final buffer (20 mM Tris-HCl pH 7.4, 0.5 M NaCl) and purity determined by SDS–PAGE.

### 2.2. Binding of HSP47 to Toolkit Peptides

The peptides of the collagen II and III toolkits were synthesized on TentaGel R Ram resin using a CEM Liberty microwave-assisted peptide synthesizer as described previously [22,25]. Briefly, the host–guest strategy was applied, whereby the guest sequence, which is the native collagen sequence of interest, is placed between (GPP)_5_ hosts, which are the flanking sequences that impart triple-helical conformation on the whole peptide; thus, toolkits II and III encompassed the entire collagen domains of human collagens II and III, respectively. Binding of HSP47 to the toolkit peptides was determined colorimetrically by solid-phase binding assay (SPBA). Peptides were coated at 10 µg/mL overnight at 4 °C on Immulon 2HB 96-well plates (Thermo Scientific, Loughborough, UK) and blocked for 1 h with 175 µL of binding buffer (TBS containing 5% BSA) before the addition of 100 µL of binding buffer containing 1 µg/mL recombinant HSP47 for 1 h at room temperature. Wells were washed five times with 200 µL of binding buffer (TBS containing 0.1% BSA *w*/*v*). Then, binding buffer was added containing HRP (horseradish peroxidase)-conjugated anti-His antibody at 1:1000 dilution, followed by incubation for 1 h at room temperature. After washing, color was developed using a Pierce TMB (3,3′,5,5′-tetramethylbenzidine) Substrate Kit (Thermo Scientific, Loughborough, UK) according to the manufacturer’s instructions.

### 2.3. Virtual Screening of Small Inhibitors Targeting Human the HSP47/Collagen Binding Interface

As the sequence similarity between canine and human HSP47 is 99%, and residues involved in collagen binding are all conserved, the human HSP47 structure was modelled by SWISS-MODEL [26] using the canine HSP47 crystal structure (PDB ID: 3ZHA) [24] as the template. The triple-helical structure of HSP47–integrin peptide I was built using software FoldX [27] based on the structure of the HSP47–CMP complex (PDB ID: 3ZHA) and our previously reported integrin peptide I structure (PDB ID: 1Q7D). 

To select potential HSP47 inhibitors, the SPECS database (http://www.specs.net) containing structural information for approximately 100,000 chemicals (logP ≤ 5.5 and logS ≥ –5.5) was adopted for virtual screening using software Glide6.9 (www.schrödinger.com). The docking model for HSP47 was prepared using the Protein Preparation and Grid Preparation tools in the Schrödinger Maestro interface. In the grid preparation process, the default settings were adopted for the cutoff, neutralization, scaling, and dimensions of the binding pocket. The standard precision (SP) mode of Glide was used to explore favorable binding poses. Ligand conformation was allowed to be flexible, while the protein was held as a rigid structure during the docking process. After the first round of screening, 10,000 compounds with the highest scores were selected for the second round of screening using the extra precision (XP) mode of Glide, leading to the selection of ~2000 molecules. These molecules were filtered using AutoDock4.2 for binding conformation analysis [28]. The binding site on HSP47 was defined around the center of the collagen model peptide in the crystal structure (PDB ID: 3ZHA) [24], and was covered by preparing a 75 × 75 × 75 grid box with 0.375Å spacing between grid points. The Lamarckian genetic algorithm was applied to obtain the protein–ligand-binding free energies and the spatial conformations of the bound compounds. 

### 2.4. Turbidity Assay

To analyze the inhibitory activity of the compounds against HSP47, a turbidity assay was carried out as described by Thomson [17]. Collagen from calf skin (Sigma-Aldrich, Gillingham, UK, Cat No: C9791) was solubilized as follows. The collagen was dissolved in 0.01 M acetic acid and stirred at 4 ℃ for 48 h to obtain 6 mg/mL collagen solution. On the day of experiment, the collagen solution was diluted by the reaction buffer (PBS: 20 mM phosphate buffer/50 mM NaCl, pH 7.4) on ice and then added to each well of a 96-well plate on ice. HSP47 protein together with compounds was then added to give the final HSP47 concentration range of 1–5 μΜ and the final collagen concentration range of 0.2–1.2 mg/mL. Turbidity was measured at 313 nm for a period of about 90 min at 34 ℃. The IC_50_ value for each compound used to block the HSP47–collagen interaction was determined by nonlinear regression using GraphPad Prism version 6.01 software (San Diego, CA, USA, 2012). 

### 2.5. Binding Affinity Measurement of HSP47 with Small Molecular Inhibitor

The binding affinity between HSP47 and small molecular inhibitors was assessed using the label-free microscale thermophoresis (MST) assay. The concentration of HSP47 was kept at 1 μM, while the concentration of the compound Hs1 varied between 0.15 and 78 μM. The assay was performed in PBS buffer containing 0.05% Tween-20. The final concentration of DMSO in the assay was 5%. After a short incubation period, the samples were loaded into MST NT.115 label-free standard capillaries and the MST analysis was performed using the MST device (Monolith NT.115). Concentrations on the x-axis are plotted in μM. 

### 2.6. Molecular Docking of Small Inhibitors on HSP47

The binding modes between HSP47 and the small-molecule inhibitors were analyzed using AutoDock 4.2 software. The same parameters were used as in the virtual screening. The results showed that Col003 and its analogs and the compounds reported by others preferred binding to the large subpocket1 near Asp385 on HSP47; However, for compound Hs1, docking showed part of the molecule binding to the small subpocket 2 close to Arg222 on HSP47. As such, we used AutoDock Vina software [29] to confirm the docking results. The binding site on HSP47 was also centered on the collagen model peptide in the crystal structure (PDB: 3ZHA), the search spaces were set as 30Å, 30Å and 30Å on x, y and z axes, and other parameters were set at default values. The binding conformations of the ligands for HSP47 were selected for further analysis by taking account of both the predicted binding free energy and the number of conformations identified.

## 3. Results and Discussion

### 3.1. Binding of HSP47 to the Collagen Toolkit Peptides

To identify the HSP47 binding site on native collagens II and III, we screened HSP47 binding sites on collagen using the collagen toolkits II and III, respectively. These kits contained sets of overlapping triple-helical peptides (THPs), as briefly described above [22]. Each peptide contains a guest sequence of 27 amino acids, the C-terminal 9 amino acids of which form the first 9 guest amino acids of the next peptide; thus, the toolkit advances 18 amino acids along the triple-helical sequence of human collagen II or III with each successive peptide, and a 9-amino acid overlap is included between adjacent peptides. These kits have been successfully used to identify many ligand-binding motifs on collagen [23,30,31]. Recombinant HSP47 with a C-terminal His-tag was incubated in wells of Immulon 2HB 96-well plates coated with toolkit peptides. Certain integrin-binding collagen peptides (GX^−4^O^−3^GE^−1^R^0^) with an Arg at position Y^0^ and different residues at position X^−4^ were selected as control peptides [32,33,34].

**Figure 1:**
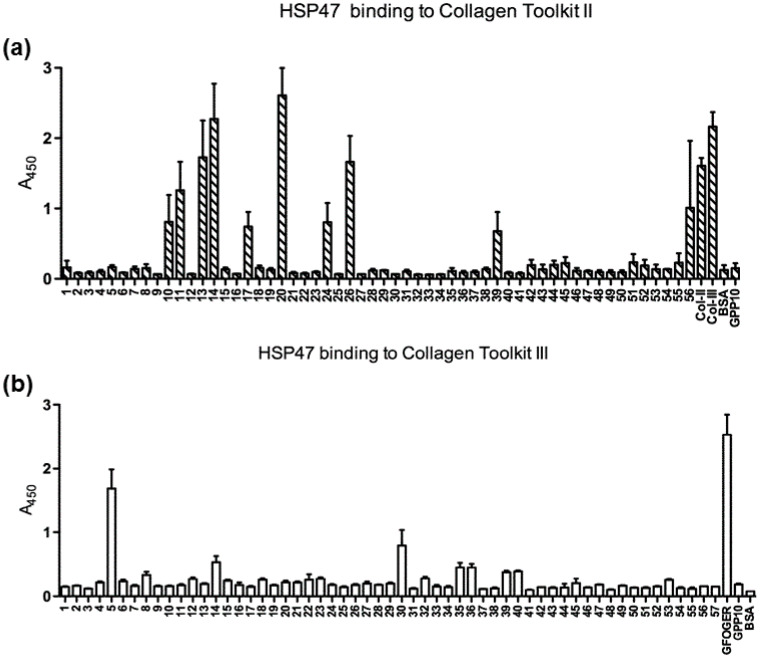
Binding of recombinant HSP47 to collagen toolkits in coated 96-well plates was measured as described in Material and Methods. (**a**) shows binding to toolkit II with collagens II and III as positive controls as indicated, and (**b**) to toolkit III, with GFOGER as positive control. BSA and GPP10 served as negative control surface coatings. Data are the means + S.E. of three independent experiments.

Significant binding of HSP47 could be detected from 12 peptides, 9 derived from collagen II and 3 from collagen III (Table 1, Figure 1). Amongst them, four collagen II peptides (II-13, II-14, II-20, II-26) and one collagen III peptide (III-5) showed stronger binding signals. Interestingly, most of these peptides are located near the N-terminus of collagen. As the triple helix assembly of procollagen occurs from the C-terminus to the N-terminus, HSP47 is likely to play a stabilization role only at the late stage of procollagen triple helix assembly. All of these identified HSP47-binding peptides contain the Gly-Xaa-Arg or GXR motif, which is consistent with previous findings that the arginine residue in these THPs is critical for HSP47 binding; however, there are more than 40 GXR motifs in both collagens II and III, only 12 of which showed appreciable HSP47 binding (Figures S1 and S2). Notably, four of the five integrin-binding peptides containing GXOGER motifs showed strong HSP47 binding, while HSP47 binding on the fifth peptide with R at the X position was relatively weak (Table 1). Together, these observations indicate that sequences adjacent to the GXR motif also play an important role in defining an efficient HSP47 binding site. This is consistent with previous observations that residues located at position -3 of collagen also affected the binding of HSP47 [31]. It should also be noted that the parent toolkit peptides that contain the same HSP47-positive, integrin-binding GXOGER motifs do not generally support HSP47 binding. Peptides II-7/8, II-18/19, II-28, and II-31 contain GLOGER, GAOGER, GFOGER, and GMOGER, respectively, but show negligible HSP47 binding. The same applies to peptides III-4, III-8, III-29, and III-31. This indicates that nearby residues (-6 and -7) may restrict HSP47 binding, as discussed further below.

### 3.2. Binding Mechanism Analysis between HSP47 and Native Collagen Peptides

**Figure 2:**
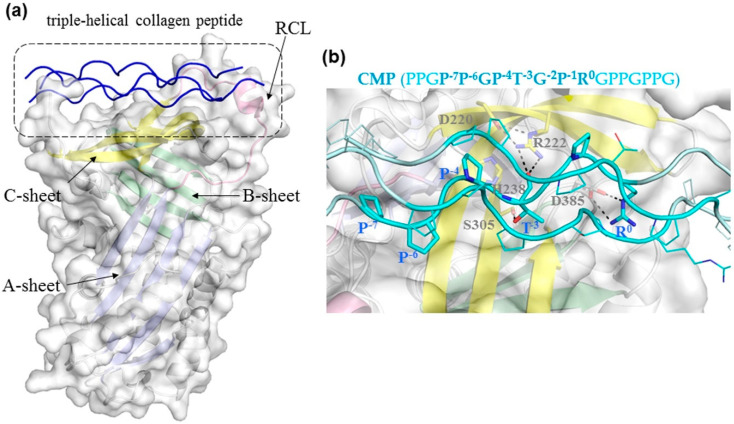
A model of human HSP47 complexed with triple-helical collagen peptide. (**a**) The model was derived from the crystal structure of canine HSP47 complexed with collagen model peptide (CMP; PDB: 3ZHA [24]). HSP47 is shown in cartoon with three beta-sheets (A, B, C) colored in pale blue, green, and yellow, respectively, while its reactive center loop is in pink. The surface of HSP47 is shown in pale grey. The triple-helical CMP is shown in blue. (**b**) Close-up of the interaction between human HSP47 and the collagen peptide (PPGP^−7^P^−6^GP^−4^T^−3^GP^−1^R^0^GPPGPPG, cyan cartoon and lines). Residues of both HSP47 and CMP that are involved in interactions are shown as sticks.

To elucidate the structural basis underlying the specific binding of these collagen peptides with HSP47, we analyzed their binding characteristics through structural modelling and binding energy calculations. A model of human HSP47 was built based on the reported crystal structure of canine HSP47 complexed with the triple-helical CMP (PPGP^−7^P^−6^GP^−4^T^−3^GP^−1^R^0^GPPGPPG, the key arginine residue is numbered as position 0). According to the crystal structure of the HSP47 and CMP complex, there are two copies of HSP47 molecules binding to a triple-helical collagen peptide in the asymmetric unit cell (PDB ID: 3ZHA). Each HSP47 molecule interacts mainly with one strand of the collagen and these two HSP47 molecules have similar binding interactions with the corresponding collagen strands [24]; therefore, the binding interactions between HSP47 and one strand of collagen were selected for subsequent analysis. The binding sites of CMP on human and canine HSP47 are almost identical, as human and canine HSP47 are highly similar, with only a few different residues. The CMP is 18 residues long, only 8 of which (P^−7^P^−6^GP^−4^T^−3^GP^−1^R^0^) are involved in direct interactions with the β-sheet C region (yellow-colored) of HSP47 (Figure 2). The Arg^0^ in the collagen formed a salt bridge to the conserved residue Asp385 in HSP47 (Figure 2b), which was essential for collagen binding, as confirmed by mutagenic study [24].

Based on this model, we then analyzed the structure–activity relationship of the binding between collagen peptides and HSP47 using FoldX software, which is a commonly-used protein stability prediction algorithm that can estimate changes in protein folding free energy caused by residue variations [27]. We applied FoldX to analyze single residue variations at different positions in CMP to estimate changes of the folding free energy compared to the WT complex. A lower folding free energy change (ΔΔG) indicates favorable binding of HSP47 and CMP. Here, we first validated this method by calculating the binding energies of a set of collagen model peptides with variations at position -3 presented in a previous report, where the bioactivities of these peptides were measured [31]. This showed that the calculated energy scores of these peptides are largely consistent with the HSP47 binding activities (Figure S3), where the most optimal residue at position -3 is Pro or Thr. Even though the hydroxyl group and the main chain of Thr^−3^ formed polar interactions with residue Arg222 and Ser305 seen in the crystal structure of HSP47 (Figure 2b), Pro at this position could bind into the same pocket and has similar binding activities [31]. Other residues such as Hyp and Met at position -3 seemed compatible with HSP47 binding as well (Figure S3), while bulky or charged residue such as Phe, His, Arg, Trp, and Tyr were not. This is consistent with the HSP47 binding peptides identified here from the toolkits with Pro, Hyp, Met (III-14), or Thr (II-10) at position -3 (Table 1). 

**Figure 3:**
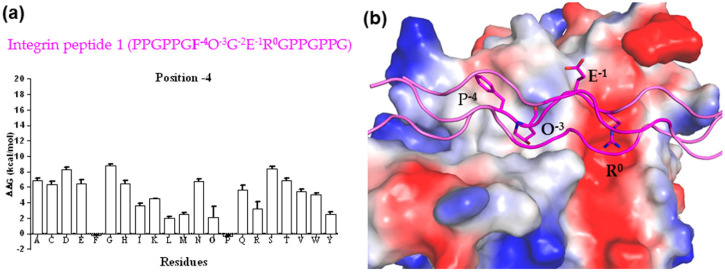
(**a**) Predicted total folding energies for the substitution of residues at position -4 in integrin peptide 1 (PPGP^−7^P^−6^GF^−4^O^−3^GE^−1^R^0^GPPGPPG) and the HSP47 complex compared to that of WT (ΔΔG ± SD, n = 5, kcal/mol). The lower energy indicates favorable binding. (**b**) The predicted binding mode between triple-helical integrin peptide 1 (shown as magenta cartoon and sticks) and HSP47 (shown on electrostatic potential surface).

Subsequently, FoldX was applied to analyze the impact of substitutions at position -4 on the HSP47-collagen binding energy. We found that Pro and Phe at this position bind better than Hyp, Leu, Met, and Ala, while molecular modelling of the integrin peptide 1 (GF^−4^OGER^0^) showed that Phe at position -4 could fit into a shallow surface cleft of HSP47, providing potential stabilizing interactions (Figure 3). Smaller hydrophobic residues such as Met and Leu seen in other integrin peptides could also be accommodated in this surface cleft, while the charged bulky side chain of an arginine residue could not. This readily explains why HSP47 binds tightly on integrin peptide 1 but very poorly on integrin peptide 5 (Table 1); however, there were differences between the experimental and predicted results for Ala in position -4, which was likely an outlier from the calculation by FoldX (Table 1, Figure 3).

We also assessed the effects of substitutions at positions -7 and -6 in CMP (PPGP^−7^P^−6^GP^−4^T^−3^GP^−1^R^0^GPPGPPG), which are located at the other end of the HSP47 binding site on collagen. It appeared that all the non-Pro substitutions showed worse binding to HSP47 (Figure S4). This may be due to these positions being far away from the key anchoring Arg^0^, while replacement of proline, which has a rigid sidechain, will increase the flexibility in this part of the triple-helical conformation, leading to lower binding activities of these peptides. Although not all of the unbound peptides with the GXR motif in toolkits II and III could be readily explained by the simple free folding energy calculation (Figure S2), the binding activities of collagen peptides towards HSP47 most likely reflect the overall cumulative effects of all of the residues involved in the HSP47–peptide binding interface. Arg^0^ is the most critical residue in collagen, but residues at positions -3 and -4 of collagen peptides play a critical supporting role for efficient HSP47 binding. This indicates that targeting the binding surfaces of HSP47 corresponding to positions -4, -3, and 0 of collagen simultaneously would provide a more efficient approach to blocking HSP47 activity than targeting the binding surface of HSP47 at position 0 only. 

### 3.3. Screening and Identification of HSP47 Small Molecular Inhibitors 

**Figure 4:**
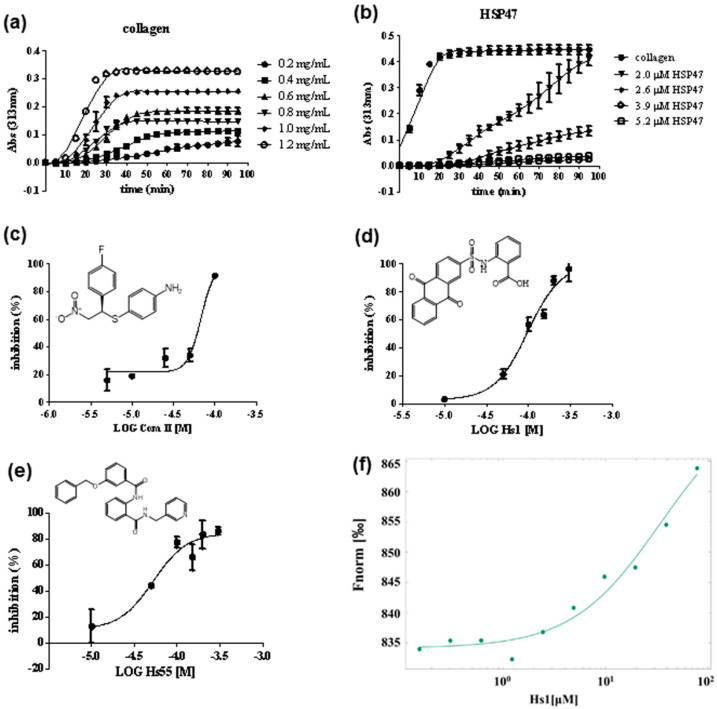
Screening and discovery of HSP47 small molecular inhibitors. Collagen fibril formation was measured in the absence (**a**) and presence (**b**) of HSP47. Collagen fibril formation was monitored at 313 nm in phosphate buffer. Inhibition effect of Com II (**c**), Hs1 (**d**), and Hs55 (**e**) against HSP47 in turbidity assay. (**f**) The binding activity of Hs1 against HSP47 in the label-free MST experiment.

The binding mode between HSP47 and the collagen peptide shown above was used to design a virtual screening for potential HSP47 inhibitors, focusing on the footprint of collagen residues 0, -3, and -4 upon the surface of HSP47. After initial screening, we manually selected 58 compounds for subsequent inhibitory activity assay according to the diversity and potential interactive mechanisms between the compounds and HSP47. Collagen molecules in a buffer of physiological pH spontaneously associated to form fibrils [17,35,36], while the turbidity gradually increased in a concentration-dependent manner and then reached a plateau phase (Figure 4a). HSP47 retarded this fibril formation process within the range of 2.0–5.2 μM (Figure 4b). An HSP47 concentration of 2.6 μM was used for the compound activity assay (Figure 4c–e), while a previously reported HSP47 inhibitor, Com II, was selected as a positive control. 

Our measurement showed IC_50_ values for Com II in the range of 50–100 μM (Figure 4c), which was comparable to the reported value of 26.6 μM [17]. Amongst our selected compounds, compound Hs1 and Hs55 have the most promising activity, with IC_50_ values of 97 μM and 55 μM, respectively (Figure 4d,e). As Hs1 had better water solubility, we determined its binding affinity to HSP47 using microscale thermophoresis (MST). This revealed that Hs1 binds human HSP47 with a dissociation constant (Kd) of ~33 μM (Figure 4f), consistent with its blocking activity in inhibiting collagen fibril formation (Figure 4d).

### 3.4. Binding Modes of Small Molecular Inhibitors on HSP47

**Figure 5:**
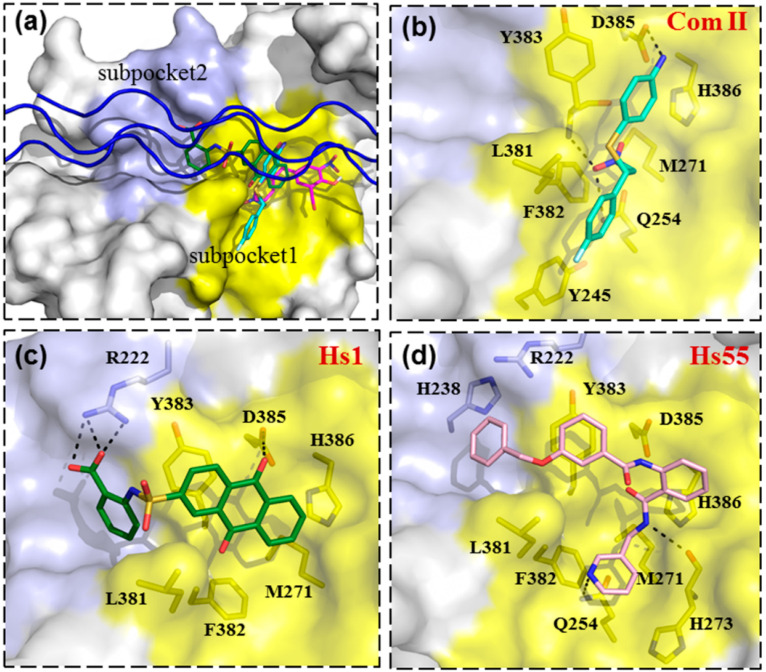
Predicted binding mode of HSP47 inhibitors. (**a**) HSP47 is shown as surface presentations, while the collagen mode peptide (blue) is shown as a cartoon. The Arg0 binding area on HSP47 is colored yellow (subpocket 1). while the binding area for collagen residues at positions -4 and -3 is colored slate (subpocket 2). The potential binding interactions between HSP47 and the compounds Col003 (**a**) COM II (**b**), Hs1 (**c**), and Hs55 (**d**) are shown in the compounds as sticks and labeled. Compounds Col003, Com II, Hs1, and Hs55 are shown as magenta, cyan, green, and pink sticks, respectively. The black dashed lines represent the hydrogen bonds.

To predict how these inhibitors would bind on HSP47, we docked these molecules on HSP47 using AutoDock4 and AutoDock Vina software. The docking grid box was defined to include the whole collagen binding region on the HSP47 surface. Apart from Com II, Hs1, and Hs55, a recently identified HSP47 inhibitor Col003 and its analogues were also selected for docking [20,36]. The docking results indicated that Com II and Col003 bind to the surface area of HSP47 involved in binding the GXR motif of collagen (colored yellow in Figure 5, subpocket1). For Com II, the nitrogen atom of the aniline group forms a hydrogen bond with Asp385, while the NO_2_ group forms a hydrogen bond with Gln254 and the main chain of Tyr383, which might play an essential role in the compound binding. The fluorophenyl group of Com II stacks on the relatively hydrophobic area of HSP47 formed by Tyr245, Met271, Leu381, and Phe382. Col003 binds to a similar position, forming hydrogen bonds with His274 and the main chain of His273 of HSP47 through its hydroxy group, also forming a hydrogen bond with the main chain of Val275 through the carbonyl group (Figure 5a and Figure S5). Its benzyl moiety binds the same hydrophobic patch of HSP47 as Com II (Figure 5b and Figure S5). In contrast, the binding of both Hs1 and Hs55 seems to involve both subpocket1 (Figure 5a) and the neighboring surface area for the docking of collagen residues at positions -3 and -4 (pale blue, subpocket2, Figure 5a), mimicking a collagen peptide chain binding both these surface pockets simultaneously. The carboxyl group of Hs1 forms hydrogen bonds with Arg222 of HSP47, while the hydroxyl group on 9,10-dihydroanthracene forms a hydrogen bond with Asp385. At the same time, the 9,10-dihydroanthracene ring forms hydrophobic interactions with Met271, Leu381, Phe382, and Tyr383. Hs55 has a longer extended conformation and docks in a similarly orientation as Hs1, bridging both collagen binding pockets of HSP47 simultaneously. Although the binding mode of these compounds on HSP47 needs to be verified by structural studies, these docking results indicate that it is feasible to target two subpockets on the HSP47 surface at the same time. Subsequent chemical structure modification may improve the activities and specificity of these compounds for efficient HSP47 activity inhibition.

## 4. Conclusions

As HSP47 is an attractive therapeutic target for the treatment of fibrotic diseases. Understanding of its physiological binding interactions would provide valuable information for the selection and design of small HSP47 inhibitors. Here, we identified the HSP47 binding sites on native collagens II and III using the collagen toolkits and showed that collagen binding involves two neighboring subpockets on the HSP47 surface. Subsequent structure-based drug screening through targeting of these surface areas identified two novel small-molecule HSP47 inhibitors, which might serve as lead compounds for the development of HSP47 inhibitors through chemical structure modification.

## Figures and Tables

**Table 1:** Identification of HSP47-binding THPs from the toolkits II and III. *GPP* represents the start or finish of the [*GPP*]_5_ host peptide, while O represents hydroxyproline (Hyp). The GXR region that is predicted to play a key role in HSP47 binding is colored red. For the sequences that contain more than one GXR motif, we highlighted the most plausible one according to our energy prediction results (Figure S4c). The integrin-binding THPs with a GXOGER sequence flanked by [*GPP*]_5_ at both ends, where X (colored blue in the table) represents F, A, M, L, or R, were used as controls.

Peptide Name	Sequence	Mean A_450_
**Toolkit II**		
14	---------------------------------------*GPP***GPR**GPOGPQGATGPLGPKGQTGEOGIA *GPP*	>2
20	------*GPP*GANGDOGROGEOGLO**GAR**GLTGROGDA*GPP*	>2
13	-*GPP*GAKGSAGAOGIAGAOGFO**GPR**GPOGPQ*GPP *	1.5–2
26	---------------------------------------*GPP***GER**GEQGAOGPSGFQGLOGPOGPOGEG*GPP*	1.5–2
11	--------------------*GPP*GARGPEGAQ**GPR**GEOGTOGSOGPAGAS*GPP *	1–1.5
10	GPPGGOGFOGAOGAKGEAGPT**GAR**GPEGAQ*GPP*	0.5–1
17	- GPPGKRGARGEOGGVGPIGPO**GER**GAOGNR*GPP*	0.5–1
24	-------------*GPP*GKAGEKGLOGAO**GLR**GLOGKDGETGAA*GPP*	0.5–1
39	---------------------------------------*GPP***GAR**GAQGPOGATGFOGAAGRVGPOGSN*GPP*	0.5–1
**Toolkit III**		
5	---------------------------------------*GPP***GER** GLOGPOGIKGPAGIOGFOGMKGHR*GPP*	1.5–2
30	--------------------------------*GPP*GAO**GLR**GGAGPOGPEGGKGAAGPOGPO*GPP*	0.5–1
14	--------------------*GPP*GIOGAOGLM**GAR**GPOGPAGANGAOGLR*GPP*	0.5–1
**Integrin Peptide**		
1	*---------------------------------GPP*G**F**O**GER***GPP*	>2
2	*--------------------------------GPP*G**M**O**GER***GPP*	1.5–2
3	*---------------------------------GPP*G**A**O**GER***GPP*	1.5–2
4	*---------------------------------GPP*G**L**O**GER***GPP*	1.5–2
5	*---------------------------------GPP*G**R**O**GER***GPP*	0–0.5

## Supplementary Materials

The following are available online at https://www.mdpi.com/article/10.3390/biom11070983/s1.
