# Supplementary material for: Identification of HSP47 Binding Site on Native Collagen and Its Implications for the Development of HSP47 Inhibitors
Source: Biomolecules. 2021 Jul 3;11(7):983. doi: 10.3390/biom11070983 (PMC8301893; doi:10.3390/biom11070983)
Supplement: Supplementary file 1 [file biomolecules-11-00983-s001.zip › biomolecules-1274422-supplementary.pdf]

Supplementary Materials

(**a**)

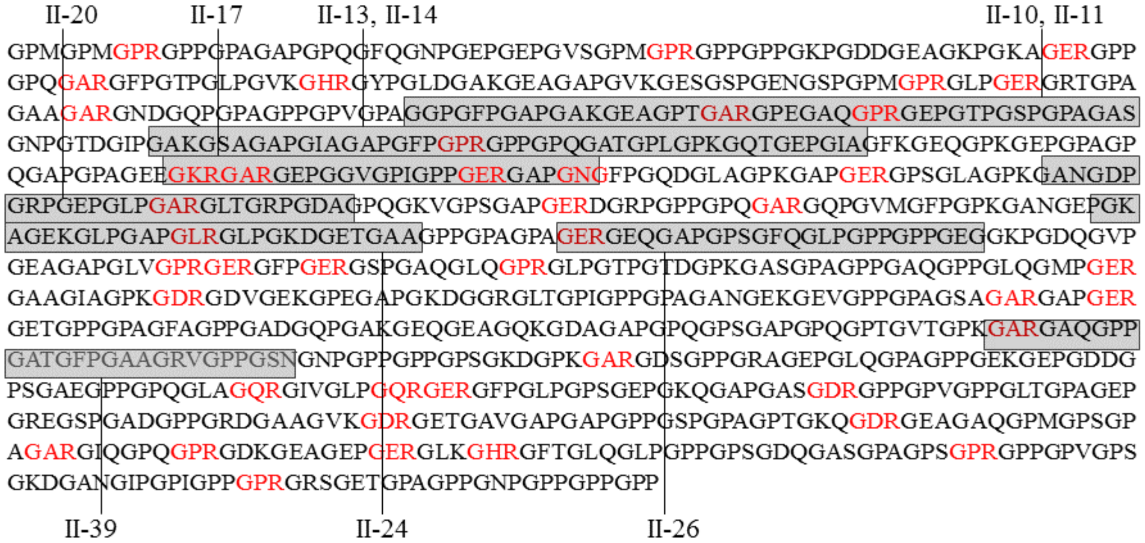

(**b**)

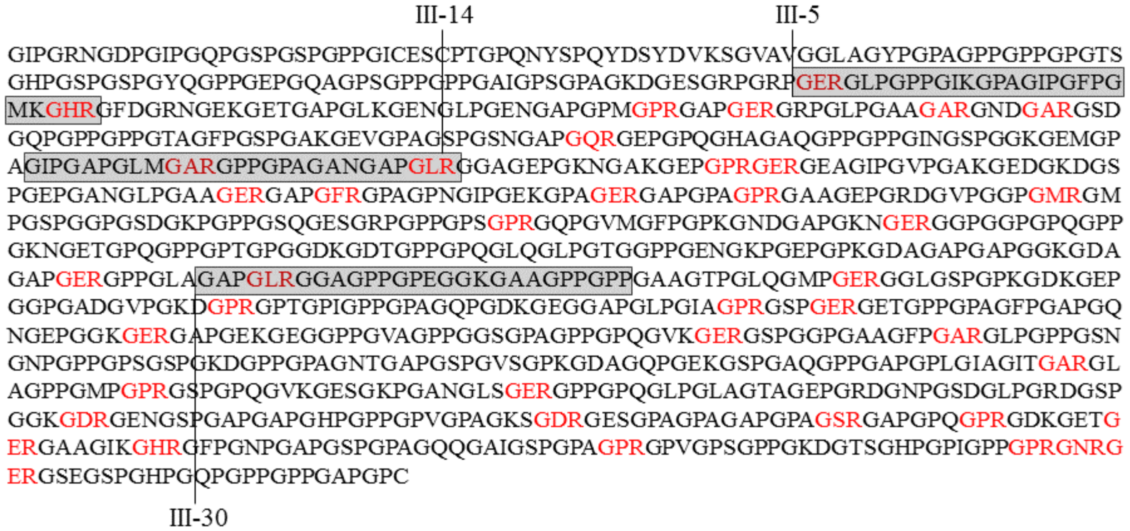

Figure S1. Binding sites of HSP47 on collagen II (**a**) and collagen III (**b**) with the corresponding collagen sequences highlighted. All the GXR sequences are colored red. The sequences do not include post-translational modification of proline (P).

(a)

	GX-1R0	
GPMGPM	**GPR**	GPOGPAGAOGPQGFQGNO
GPQGFQGNOGEOGEOGVSGPM	**GPR**	GPO
GPM	**GPR**	GPOGPOGKOGDDGEAGKOGKA
GEAGKOGKA	**GER**	GPOGPQGARGFOGTP
	**GAR**	GFOGTOGLOGVKGHRGYOGLDGAK
GESGSOGENGSOGPM	**GPR**	GLOGERGRT
GLO	**GER**	GRTGPAGAAGARGNDGQOGPA
GEOGPAGPQGAOGPAGEE	**GKR**	GARGEO
GKRGARGEOGGVGPIGPO	**GER**	GAOGNR
	**GER**	GAOGNRGFOGQDGLAGPKGAOGER
GPKGAO	**GER**	GPSGLAGPKGANGDOGRO
GAOGEDGROGPOGPQ	**GAR**	GQOGVMGFO
GKDGETGAAGPOGPAGPA	**GER**	GEQGAO
GEAGAOGLVGPRGERGFO	**GER**	GSOGAQ
	**GER**	GSOGAQGLQGPRGLOGTOGTDGPK
GAQGPOGLQGMOGERGAAGIAGPK	**GDR**	
GIAGPK	**GDR**	GDVGEKGPEGAOGKDGGR
GAOGKD	**GGR**	GLTGPIGPOGPAGANGEK
GPAGANGEKGEVGPOGPAGSA	**GAR**	GAO
GSAGARGAO	**GER**	GETGPOGPAGFAGPO
GPSGAOGPQGPTGVTGPK	**GAR**	GAQGPO
GPSGKDGPK	**GAR**	GDSGPOGRAGEOGLQ
GLAGQRGIVGLOGQR	**GER**	GFOGLOGPS
GFOGLOGPSGEOGKQGAOGAS	**GDR**	GPO
GAS	**GDR**	GPOGPVGPOGLTGOAGEOGRE
GRDGAAGVK	**GDR**	GETGAVGAOGAOGPO
GAOGAOGPOGSPGPAGPTGKQ	**GDR**	GEA
GKQ	**GDR**	GEAGAQGPMGPSGPAGARGIQ
GPAGARGIQGPQGPRGDKGEAGEO	**GER**	
GEAGEO	**GER**	GLKGHRGFTGLQGLOGPO
GLQGLOGPOGPSGDQGASGPAGP S	**GPR**	
GPAGP S	**GPR**	GPOGPVGPSGKDGANGIO
GKDGANGIOGPIGPO	**GPR**	GRSGETGPA
	**GPR**	GRSGETGPAGPOGNOGPOGPOGPO

(b)

	GX-1R0	
GPSGPAGKDGESGROGRO	**GER**	GLOGPO
GFOGMK	**GHR**	GFDGRNGEKGETGAOGLK
GETGAOGLKGENGLOGENGAOGPM	**GPR**	
GAOGPM	**GPR**	GAOGERGROGLOGAAGAR
GLOGAA	**GAR**	GNDGARGSDGQOGPOGPO
GAKGEVGPAGSOGSNGAO	**GQR**	GEOGPQ
	**GQR**	GEOGPQGHAGAQGPOGPOGINGSO
GANGAOGLRGGAGEOGKNGAKGEO	**GPR**	
GAKGEO	**GPR**	GERGEAGIOGVOGAKGED
GANGLOGAA	**GER**	GAOGFRGPAGPNGIO
GPAGPNGIOGEKGPAGERGAOGPA	**GPR**	
GAOGPA	**GPR**	GAAGEOGRDGVOGGOGMR
GVOGGO	**GMR**	GMOGSOGGOGSDGKOGPO
GSDGKOGPOGSQGESGROGPOGPS	**GPR**	
GPOGPS	**GPR**	GQOGVMGFOGPKGNDGAO
GPKGNDGAOGKN	**GER**	GGOGGOGPQGPO
GGKGDAGAOGERGPOGLAGAO	**GLR**	GGA
GAAGPOGPOGAAGTOGLQGMO	**GER**	GGL
GMO	**GER**	GGLGSOGPKGDKGEOGGOGAD
GEOGGOGADGVOGKD	**GPR**	GPTGPIGPO
GEGGAOGLOGIAGPRGSO	**GER**	GETGPO
	**GER**	GETGPOGPAGFOGAOGQNGEOGGK
GQNGEOGGK	**GER**	GAOGEKGEGGPOGVA
GPOGPQGVK	**GER**	GSOGGOGAAGFOGAR
GAAGFO	**GAR**	GLOGPOGSNGNOGPOGPS
GAOGPLGIAGITGARGLAGPOGMO	**GPR**	
GPOGMO	**GPR**	GSOGPQGVKGESGKOGAN
GESGKOGANGLS	**GER**	GPOGPQGLOGLA
GNOGSDGLOGRDGSOGGK	**GDR**	GENGSO
	**GDR**	GENGSOGAOGAOGHOGPOGPVGPA
GPOGPVGPAGKS	**GDR**	GESGPAGPAGAO
GPAGPAGAOGPAGSRGAOGPQ	**GPR**	GDK
GPQ	**GPR**	GDKGETGERGAAGIKGHRGFO
GIK	**GHR**	GFOGNOGAOGSOGPAGQQGAI
GPAGQQGAIGSOGPA	**GPR**	GPVGPSGPO
GPVGPSGPOGKDGTSGHOGPIGPO	**GPR**	
GPIGPO	**GPR**	GNRGERGSEGSOGHOGQO
	**GER**	GSEGSOGHOGQOGPOGPOGAOGPC

Figure S2. Sequences of 'GXR' containing collagen peptides from Toolkits II (a**) and III (**b**). O represents hydroxyproline.** For the sequences that contain more than one 'GXR' motif, we highlighted the most plausible motif according to the calculated results from of substitution at position -1 (Figure S4c).

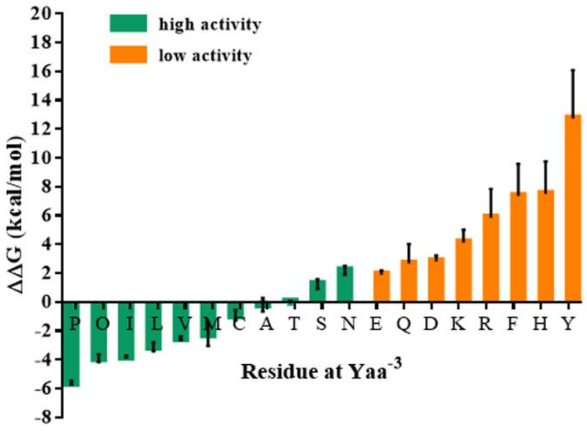

Residue at position -3 (Yaa^{-3})	Predicted folding free energy compared to WT (ΔΔG (kcal/mol))	Reported IC_{50} value (μM) [(a)]
P	-5.60±0.13	0.94
O	-3.90±0.26	4.60
I	-3.78±0.06	19.00
L	-3.10±0.31	37.00
V	-2.52±0.12	8.40
M	-2.23±0.82	43.00
C	-0.93±0.36	4.20
A	-0.18±0.46	9.30
T	0.03±0.04	0.58
S	1.24±0.34	4.00
N	2.20±0.31	41.00
E	1.89±0.31	>100
Q	2.66±1.36	>100
D	2.82±0.40	>100
K	4.13±0.88	>100
R	5.85±2.00	>100
F	7.36±2.23	79.00
H	7.50±2.24	64.00
Y	12.73±3.35	95.00

Figure S3. The predicted folding free energy of a serial of Yaa^{-3}-substituted synthetic collagen-model peptides compared to peptide 8 (WT: $POGP^{-7}O^{-6}GP^{-4}T^{-3}GP^{-1}R^{0}GPOGPO$) binding of HSP47. Peptides with reported IC_{50} values lower than 50 μM are defined as high activity peptides (green), those higher than 50 μM are defined as low activity peptides (orange).

[a] Reference. 31

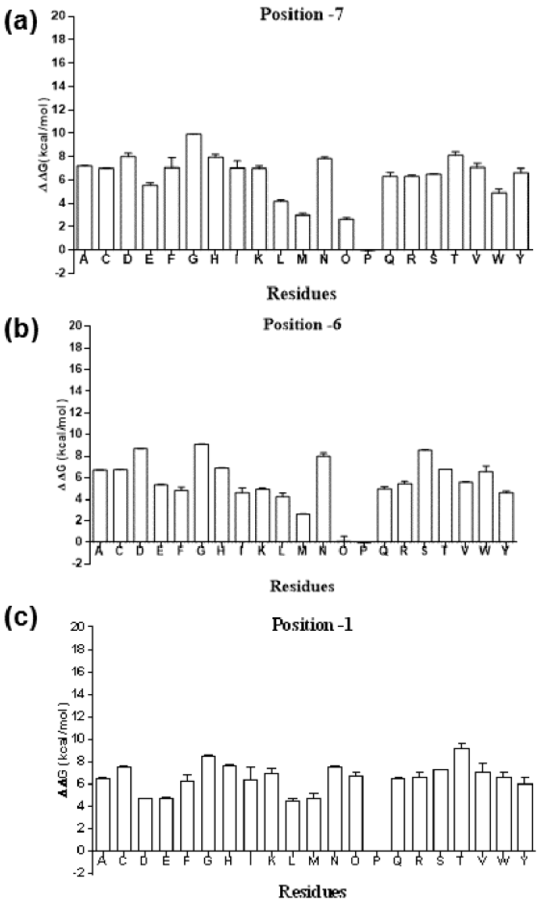

Figure S4. Predicted folding free energy changes for the substitutions at positions -7, -6 and -1 in CMP ($PPGP^{-7}P^{-6}GP^{-4}T^{-3}GP^{-1}R^{0}GPPGPPG$) on binding of HSP47. Data were shown as ΔΔG ± SD (kcal/mol) (n=5).

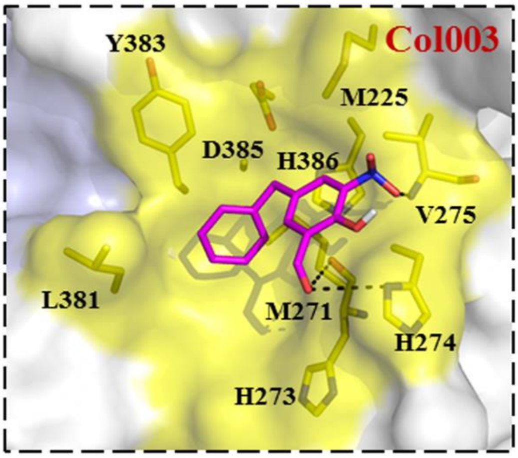

Figure S5. Predicted binding mode of Col003 to HSP47. HSP47 is shown as a colored surface. Residues of HSP47 that involved in interacting with the compounds are shown in sticks and labeled. Compound Col003 is shown in magenta sticks. The black dashed lines present hydrogen bonds.
